# Dynamic single-cell systemic immune responses in immunotherapy-treated early-stage HR+ breast cancer patients

**DOI:** 10.1038/s41523-025-00776-1

**Published:** 2025-07-03

**Authors:** Xiaopeng Sun, Margaret L. Axelrod, Adrienne G. Waks, Jingxin Fu, Molly DiLullo, Eliezer M. Van Allen, Sara M. Tolaney, Elizabeth A. Mittendorf, Yaomin Xu, Justin M. Balko

**Affiliations:** 1 Cancer Biology Program, Vanderbilt University, Nashville, TN USA; 2 Department of Medicine, Washington University in St Louis, St Louis, MO USA; 3 Department of Medical Oncology, Dana-Farber Cancer Institute, Boston, MA USA; 4 Cancer Program, Broad Institute of MIT and Harvard, Boston, MA USA; 5 Parker Institute for Cancer Immunotherapy, Dana-Farber Cancer Institute, Boston, MA USA; 6 Department of Surgery, Brigham and Women’s Hospital, Boston, MA USA; 7 Department of Biostatistics, Vanderbilt University Medical Center, Nashville, TN USA; 8 Department of Medicine, Vanderbilt University Medical Center, Nashville, TN USA; 9 Department of Pathology, Microbiology, and Immunology, Vanderbilt University Medical Center, Nashville, TN USA; 10 Breast Cancer Research Program, Vanderbilt University Medical Center, Nashville, TN USA

**Keywords:** Biomarkers, Predictive markers, Cancer, Tumour immunology

## Abstract

The limited added benefit of immune checkpoint inhibitors in breast cancer indicates the pressing need to identify biomarkers of response to minimize risk and maximize benefit. We used single cell RNA sequencing and T cell receptor (TCR) sequencing of peripheral blood mononuclear cells (for 28 samples comprising 79,284 cells) to monitor the peripheral immune dynamic of an exploratory cohort of hormone receptor positive breast cancer patients treated with neoadjuvant nab-paclitaxel+pembrolizumab with the ultimate goal of identifying potential peripheral blood predictive biomarkers. In responsive patients, Granzyme B positive (*GZMB*+) cytotoxic CD8 T cells expanded post-nab-paclitaxel+pembrolizumab, accompanied by rapid changes in TCR clones. In contrast, non-responders’ peripheral T cells may experience terminal exhaustion and are not significantly altered by treatment. In addition, B cell and monocyte-specific interferon response signatures were also associated with response. Our data suggests that peripheral immunological signatures may represent a facile way to monitor dynamic antitumor immune response.

## Introduction

Breast cancer is the most common cancer in women and the leading cause of cancer-related death in women. Notably, hormone receptor-positive (HR+)/human epidermal growth factor receptor 2 negative (HER2−) breast cancer emerges as the predominant subtype, encompassing up to 75% of cases. In the management of high-risk early-stage HR+/HER2− breast cancer, the standard of care typically involves upfront surgery followed by adjuvant systemic therapies, including endocrine therapy and, more recently, CDK4/6 inhibitors. Neoadjuvant therapy—either chemotherapy or, less commonly, hormone therapy in postmenopausal patients—may be considered in select cases, particularly those with locally advanced disease, where the goal is to reduce tumor burden and facilitate surgical resection. Traditionally perceived as immune “cold,” HR+ breast cancers were deemed less sensitive to immunotherapy, largely due to low tumor-infiltrating lymphocyte (TIL) counts and tumor mutational burden (TMB). However, recent clinical trial results demonstrated a significant increase of pathological complete response (pCR) for patients treated with immune checkpoint inhibitor (ICI) + chemotherapy combination^1–3^. Despite these promising results, the augmented efficacy of this regimen is paralleled by a high risk for immune-related toxicities, underscoring the critical need for biomarkers that select patients who will benefit from immunotherapy.

The cancer immunity cycle suggests that localized antitumor immune response cannot persist without continuous communication with the periphery^4,5^. Therefore, monitoring the systemic response through peripheral blood offers a promising and potentially convenient approach to predict patient response to ICI and understand the biological dynamics of systemic ICI response. Previous studies from our lab have demonstrated that peripheral cytotoxic gene signatures^6^ and monocyte abundance^7^ are associated with chemotherapy outcome in breast cancer. In melanoma and basal cell carcinoma, clonal expansion of peripheral effector memory T cells^8^ and clonal replacement between the peripheral and intratumoral T cell repertoires^4^ were associated with ICI-induced antitumor response. Currently, limited research has been conducted to characterize the systemic immune landscape of ICI-treated HR+ breast cancer patients. To address this gap in knowledge, we performed single-cell RNA sequencing on 28 longitudinal peripheral blood samples from seven HR+ breast cancer patients undergoing neoadjuvant nab-paclitaxel + pembrolizumab treatment as part of a clinical trial (NCT02999477). Using this approach, we identified transcriptomic signatures and specific cell populations associated with treatment and response, and provide proof of concept that these principles may be able to be extended to larger patient populations with more cost-effective technologies, such as bulk transcriptomics or targeted gene expression analyses.

## Results

### Study population

**Fig. 1:**
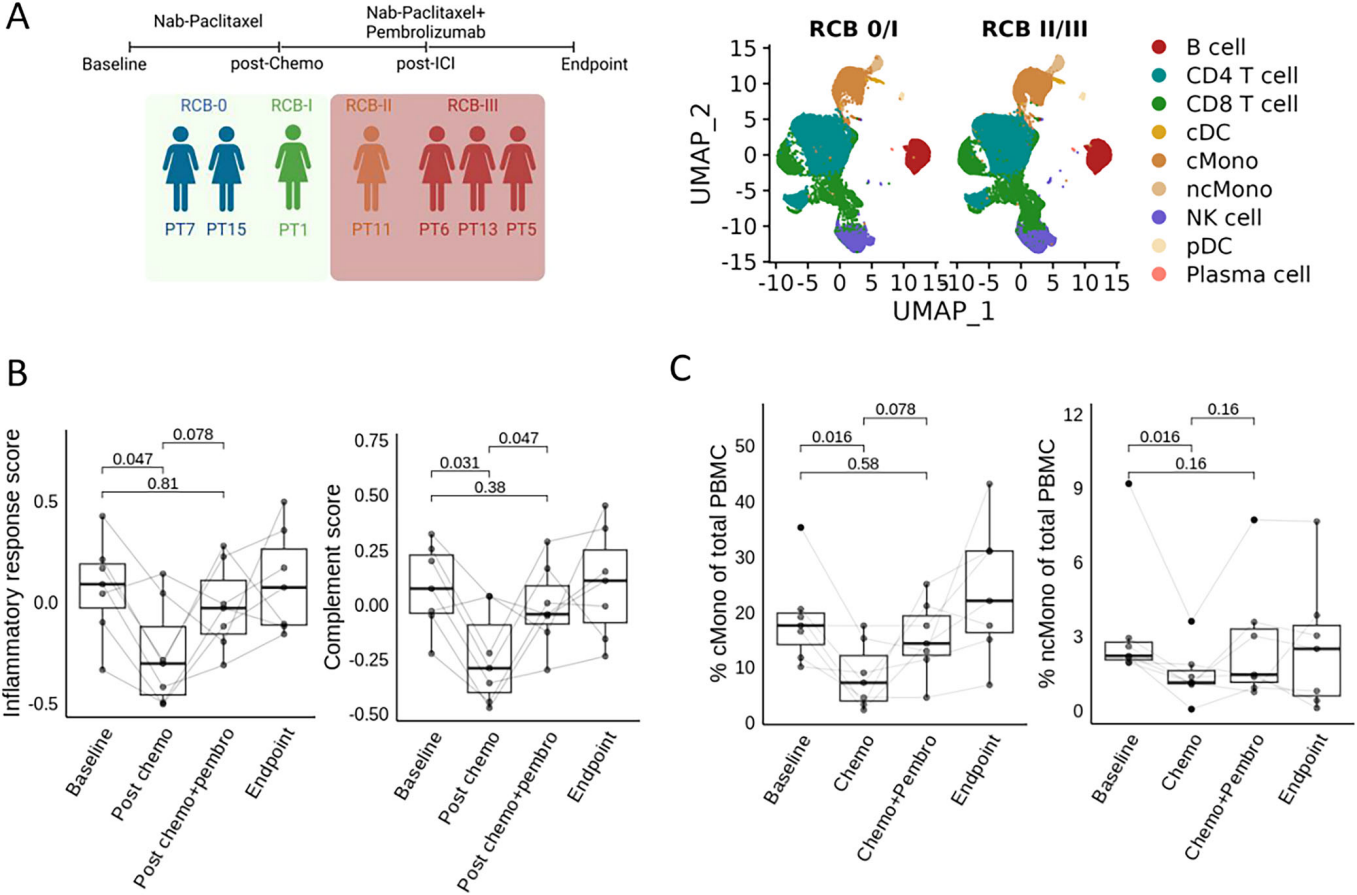
Chemotherapy induced a decrease in monocytes and inflammatory response signatures that were rescued by ICI. **A** Single-cell RNA sequencing was performed on longitudinal blood samples collected from seven patients, with nine major cell clusters identified. **B**) ssGSEA score for Hallmark Interferon response and Complement response calculated after aggregating the expression profile as a pseudobulk sample, a paired Wilcoxon test was performed. **C** The percentage of classical monocyte (left) and non-classical monocyte (right) out of the number of PBMC detected was compared across timepoints using a paired Wilcoxon test.

Single-cell RNA and TCR sequencing were performed on 28 longitudinal PBMC samples obtained from a subset of seven HR + BC patients enrolled in the DFCI 16–466 study [NCT02999477] (Fig. 1A). This exploratory cohort represents the patients enrolled in the trial’s chemotherapy-first arm (2 weeks of nab-paclitaxel followed by combination nab-paclitaxel + pembrolizumab). The patients were selected aiming to balance the responders and non-responders while also minimizing patients receiving neoadjuvant doxorubicin and cyclophosphamide, which may confound the analysis. Baseline patient and disease characteristics are shown in Table 1. Our cohort is representative of the patients enrolled in the chemotherapy-first arm [manuscript in review; citation to be added].

**Table 1:** Patient clinical pathological characteristics

Characteristic	Arm A: chemotherapy first (*n* = 7)
Age at randomization, years - Median (range)	47 (35–57)
Sex	
Female	7 (100.0%)
Race	
White	6 (85.7%)
Asian	0 (0.0%)
More than one race	1 (14.3%)
Ethnicity	
Hispanic or Latino	1 (14.3%)
Non-Hispanic	6 (85.7%)
Unknown	0 (0.0%)
Overall clinical stage*	
II	5 (71.4%)
III	2 (28.6%)
Clinical T stage	
T2	5 (71.4%)
T3	2 (28.6%)
T4	0 (0.0%)
Clinical N stage	
N0	2 (28.6%)
N1	5 (71.4%)
HR status**	
HR positive	6 (85.7%)
HR low-positive	1 (14.3%)
HER2 status	
Negative	7 (100.0%)
Histology	
Ductal	5 (71.4%)
Lobular	2 (28.6%)
Mixed ductal + lobular	0 (0.0%)
Grade	
1	1 (14.3%)
2	2 (28.6%)
3	4 (57.1%)
Unknown	0 (0.0%)

*HR* hormone receptor.

*Based on AJCC clinical anatomic staging.

**HR status is considered positive if ER or PR ≥10%. Otherwise, considered low-positive. HR-negative patients were not eligible and not enrolled.

Patients with residual cancer burden (RCB) 0 or I were classified as responders, and those with RCB II or III as non-responders, due to similar prognosis within RCB0/I and RCB II/III groups^9^. Among patients who responded to treatment, two achieved an RCB0 and were identified as basal-type cancers per BluePrint testing. Additionally, one of these two basal-type responders was further classified as having low hormone receptor expression (<10% HR+ by immunohistochemistry staining).

#### Chemotherapy induces systemic downregulation of the inflammatory response that is rescued by subsequent immunotherapy

To delineate therapy-induced systemic immune alterations, we employed pseudobulk differential gene expression analysis. This approach enabled the identification of chemotherapy-driven changes in systemic immunity while mitigating confounding effects from cell-specific alterations. After chemotherapy, the inflammatory response and complement signatures—representing genes involved in the activation of the innate immune system’s complement cascade—were significantly reduced. The addition of immunotherapy restored these signatures. (Fig. 1B). Additionally, a concomitant reduction in the abundance of classical and non-classical monocytes was noted, which rebounded after the addition of immunotherapy (Fig. 1C). Chemotherapy also elicited a significant increase in CD4 T cell population, accompanied by a minor expansion of CD8 T cells (Supplementary Fig. 1A, B). Furthermore, a significant decrease in B cell abundance was noted after the addition of immunotherapy (Supplementary Fig. 1C).

### CD8 T cell composition and transcriptomic features are associated with clinical response to nab-paclitaxel + pembrolizumab

**Fig. 2:**
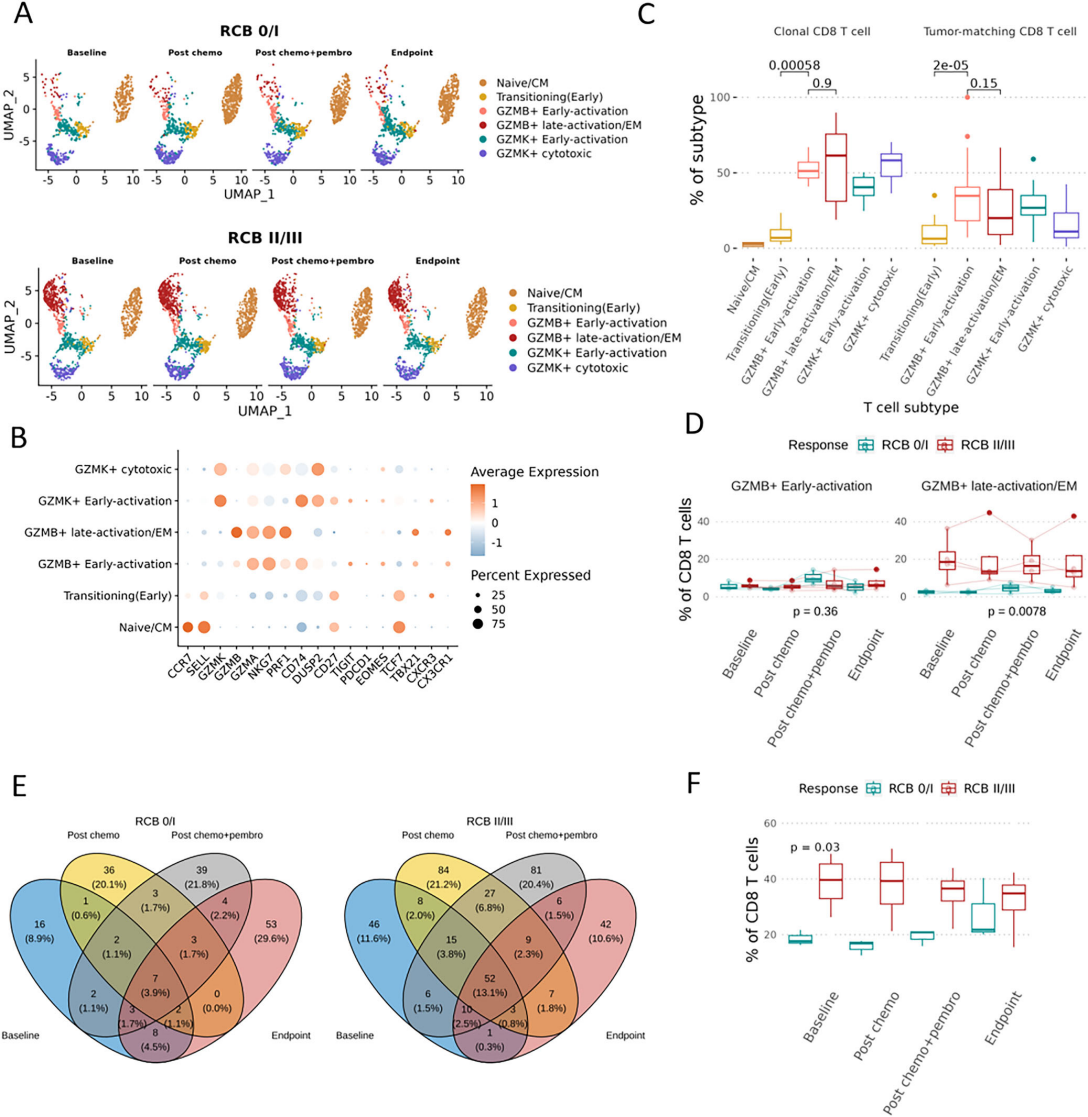
Abundance of GZMB+ late-activation/EM CD8 T cells is negatively associated with response. **A** Umap visualization of major CD8 T cell clusters, separated by the patient’s residual cancer burden after immunotherapy treatment. Responders (RCB 0/I) are depicted at the top and non-responders (RCB II/III) are at the bottom. **B** Representative gene marker for each major CD8 T cell cluster depicted in the dotplot. **C** The percentage of clonal CD8 T cells (left panel) and total CD8 T cells carrying tumor-associated TCRs (right panel) were identified. Naïve T cell does not carry any tumor-associated TCRs, and thus are not presented on the right panel. A paired Wilcoxon test was performed between T cell subtypes. **D** Abundance of CD8 T cells subtypes was compared between RCB0/I and RCB II/III patients, anova was performed. **E** Venn diagram summarizing clonal TCR overlaps across four sampling timepoints, with clonal defined as >3T cells sharing the same TCR CDR3 sequence. **F** Percentage of clonal CD8 T cells compared between RCB0/I and RCB II/III patients, anova was performed.

Given the primary role of ICI in activating T cell-mediated immunity, we examined whether the transcriptomic profile and composition of CD8 T cells were different between RCB0/I and RCB II/III patients. Counterintuitively, we observed elevated levels of classical cytotoxic markers, *GNLY, GZMB*, and *PRF1*, in patients who experienced resistance to therapy (Supplementary Fig. 2). Although the expression of cytotoxic molecules is associated with effector function (i.e., tumor cell killing), those genes were also found to be highly expressed in exhausted T cells^10^. We next analyzed whether those differentially expressed genes are contributed by a specific CD8 T cell subtype. Besides using gene expression-based markers, we also calculated the TCR CDR3 region overlaps between each T cell cluster and the TCR clones in the tumor to measure tumor association. Six CD8 T cell clusters were identified. Two GZMB+ clusters (GZMB+ early-activation and GZMB+ late-activation/effector memory-like) displayed high expression of cytotoxicity markers, high TCR clonality and strong tumor association, evidenced by the significant overlaps of their TCRs with those identified in patient-matched tumor biopsies. Two naïve clusters, including naïve/central memory-like CD8 T cells and transitioning (early) CD8 T cells displayed minimal expression of cytotoxicity genes, low clonality, and minimal tumor-match TCRs. Two GZMK+ clusters, GZMK+ early-activation CD8 T cells and GZMK+ late-activation CD8 T cells, are also clonal, tumor-associated, and exhibit signatures that resemble an intermediate stage between activation and exhaustion, reflected by their intermediate level of TCF7 and CD27 expression (Fig. 2A–C). Notably, a significantly higher proportion of GZMB+ late-activated/effector memory CD8 T cells was observed in all RCB II/III patients, which contributes to the enrichment of GZMB, PRF1, and GNLY in non-responders during differential gene expression analysis (Fig. 2D). Conversely, among RCB 0/I patients, an increase in GZMB+ early-activation CD8 T cells were noted following the addition of ICI, indicating a distinct therapy-induced immune activation (Fig. 2D and Supplementary Fig. 3).

In addition, we monitored the dynamics of CD8 T cell TCRs during treatment. Notably, RCB II/III patients exhibited a higher proportion (13%) of clonal TCRs shared longitudinally, whereas only 3.9% were maintained in RCB 0/I patients (Fig. 2E). Interestingly, the TCR repertoires of RCB 0/I patients experienced dynamic changes, with less than 15% of TCRs shared across treatments, while those of RCB II/III patients remained largely constant, with 20–40% of TCRs maintained in the blood without significant changes throughout treatment (Supplementary Fig. 4). Overall, CD8 T cells in RCB 0/I patients displayed a diverse and fluctuating TCR repertoire, with small number of clonal T cells, whereas those in RCB II/III patients were markedly more clonal (Fig. 2F). The high conversion rate of TCR in responders may indicate rapid turnover and evolution of T cells targeting tumor antigens.

### Dynamic changes in monocyte and B cell responses to nab-paclitaxel + pembrolizumab therapy

**Fig. 3:**
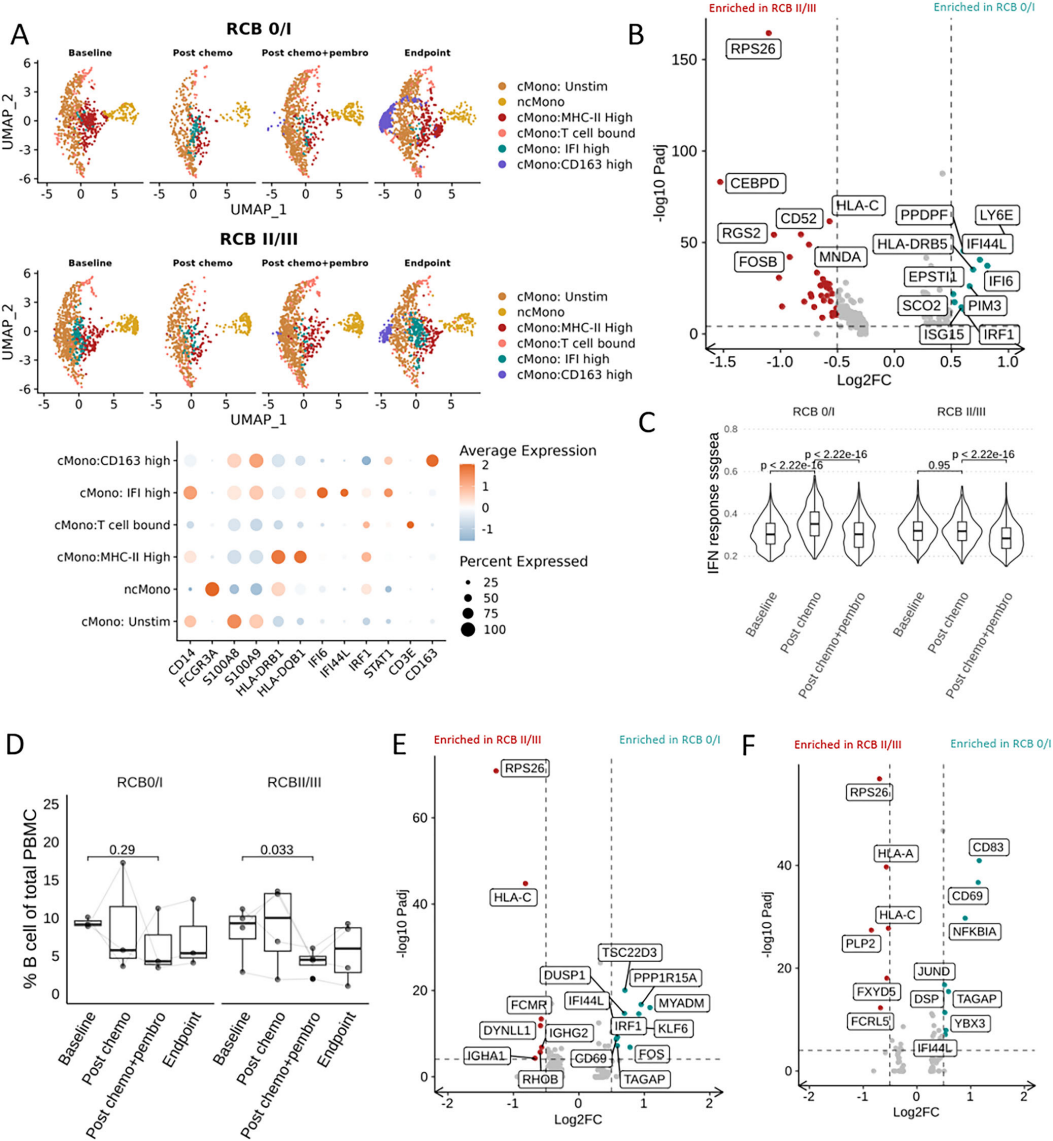
Temporal dynamics of monocytes and B cell during treatment. **A** Umap visualization of major monocyte clusters, with representative markers depicted by dotplot. **B** Differential gene expression analysis performed on total monocytes between RCB0/I and RCB II/III patients at baseline. **C** ssGSEA interferon response score calculated for individual monocytes, *t*-test was performed. **D** B cell abundance compared longitudinally, a paired *T*-test was performed. **E** Differential gene expression analysis was performed on total B cells between RCB0/I and RCB II/III patients at baseline. **F** Differential gene expression analysis performed on total B cells between RCB0/I and RCB II/III patients post-chemotherapy. For all volcano plots, Log2 fold change cutoff at 0.5, *p*adj cutoff at 1e-5.

Since we observed systemic changes in monocyte and B cell abundance during therapy (Fig. 1C and Supplementary Fig. 1C), we further compared the transcriptomic profile and composition of these antigen-presenting cells between RCB 0/I and RCB II/III patients. At baseline, monocytes from RCB 0/I patients expressed higher levels of HLA-DR and interferon response, which were further augmented by chemotherapy (Fig. 3A–C). In contrast, chemotherapy only decreased classic monocyte abundance in non-responders (Supplementary Fig. 5A). The addition of ICI induced a uniform downregulation of monocyte interferon response signal (Fig. 3C).

Responder’s B cells expressed higher levels of interferon response-related genes at baseline and following chemotherapy (Fig. 3D, E). Moreover, the expression of *CD83*, a gene associated with B cell activation and maturation, was elevated post-chemotherapy in responders compared to non-responders (Fig. 3F). The addition of ICI only significantly decreased B cell abundances in RCB II/III patients (Fig. 3D). These findings suggest the possibility of an early immune activation induced by cytotoxic chemotherapy, with monocytes and B cells potentially primed to support T cell-mediated immunity through the observed changes in their transcriptomic profiles.

## Discussion

**Fig. 4:**
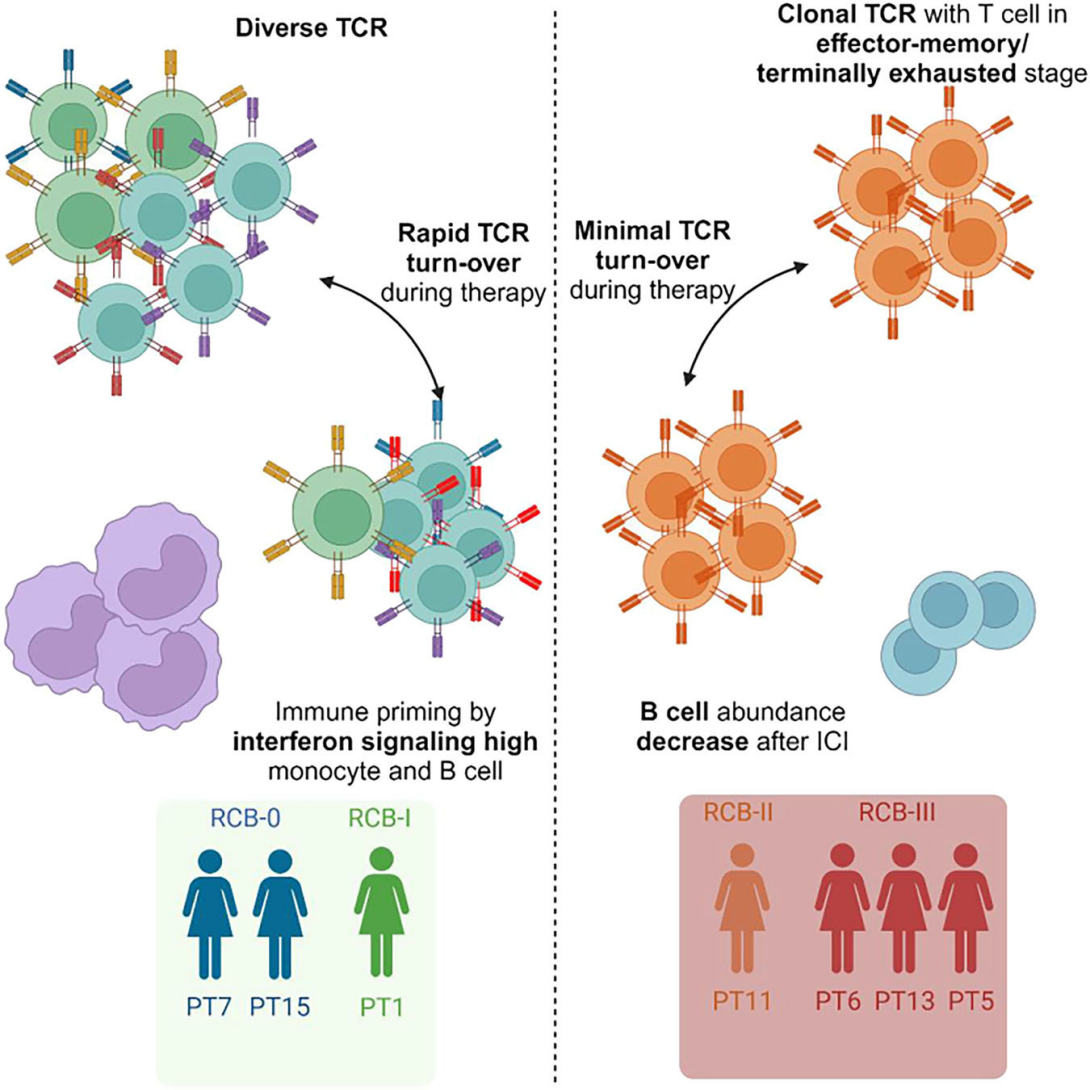
Summary of peripheral immunological features associated with Nab-paclitaxel + pembrolizumab response in HR+ breast cancer.

Peripheral immunological signatures may be useful biomarkers for immunotherapy response. In this study, we performed longitudinal single-cell analysis to examine changes in peripheral immunity in early-stage HR+/HER2− breast cancer during neoadjuvant chemotherapy and ICI treatment. Our study provides provocative evidence that the abundance of effector memory CD8 T cells, TCR clonality, and interferon response signatures in professional antigen-presenting cells could be associated with clinical response to nab-paclitaxel+pembrolizumab (Fig. 4).

T cell antitumor immunity depends primarily on their unique TCR, which recognizes the specific neoantigen presented by the major histocompatibility complex. Our analysis revealed distinct peripheral CD8 T cell dynamics between responders and non-responders. CD8 T cells from responders carry diverse TCR and experience rapid turnover during therapy, potentially due to clonal replacement^4^. Additionally, a minor expansion of GZMB+ early-activation CD8 T cells was observed among responders, consistent with previous findings where baseline abundance and post-therapy expansion of PD1 + CD8 T cells were identified as predictors of ICI response in various cancer types^11–13^. Conversely, a substantial proportion of peripheral CD8 T cells in non-responders are clonal, CX3CR1^High^, and carry effector memory features, which may have limited contribution to antitumor immunity^14^. Furthermore, the static composition of CD8 T cells in non-responders suggests that antitumor immunity may have progressed to a terminally exhausted state, potentially precluding rescue by immune checkpoint therapy^15^.

Myeloid cells have a multi-faceted role during tumorigenesis and treatment. Therefore, simply measuring neutrophil/monocyte to lymphocyte ratios may be insufficient when describing antitumor immunity. It has been proposed that chronic inflammation and the expansion of immunosuppressive myeloid cells are associated with poor response to immunotherapy^16^. Conversely, monocytes expressing migration and activation markers have been positively correlated with treatment response^11^. In our dataset, responder monocytes exhibited higher expression of HLA-DR and upregulation of interferon response genes, mirroring findings in melanoma^11^. We also observed an upregulation of interferon response signature, potentially suggesting immune activation. Interestingly, the differential interferon response signal between responders and non-responders disappeared after the addition of ICI, potentially indicating the transition from myeloid-mediated inflammation to T cell-mediated antitumor response.

Furthermore, responders displayed an enrichment of interferon response genes in baseline B cells, along with the upregulation of activation markers CD83 and CD69 post-chemotherapy. B cells in the tumor microenvironment often co-localize with other tumor-infiltrating immune cells, forming tertiary lymphoid structures and supporting antitumor immune response^17^. Additionally, serving as an antigen-presenting cell, B cell activation of T cells and generation of antibodies are key for immunotherapy response in breast cancer models^18^. While the association between peripheral B cells and their intratumoral counterparts remains unclear, the correlation between peripheral B cell abundance, B cell receptor clonality, and ICI response has been established in melanoma and NSCLC, suggesting B cells as potential biomarkers for predicting ICI response^19–21^.

Tumor biopsies study from those patients revealed that the enrichment of baseline immune activation signatures, such as interferon and inflammatory response or antigen presentation genes, are associated with responders [Clinical paper under review]. Here, we found in the blood that responders had higher baseline interferon score in B cells and upregulation of MHC-II in monocytes, potentially indicating a positive correlation between tumor microenvironment and systemic antitumor immunity. We need to further study longitudinal peripheral blood and serial tumor biopsies to understand the impact of immunotherapy on both systemic and local tumor immune environments. Due to the study’s retrospective nature and small sample size, establishing robust predictive biomarkers and causal inferences for immunotherapy response and resistance is challenging. These immunological features need validation in larger cohorts to be considered biomarkers. Despite these limitations, our findings underscore the growing interest in utilizing peripheral immunological biomarkers to predict immunotherapy response in HR+ breast cancer. With ICI becoming clinically available for TNBC and potentially HR+ breast cancer, there is an urgent need to develop a biomarker-based approach to identify patients who would benefit from therapy while minimizing toxicity. Currently, several biomarkers, such as PD-L1 expression, tumor mutational burden, tumor-infiltrating lymphocytes (TILs), and imaging modalities, have been studied in this context, albeit primarily showing prognostic utility, rather than a predictive role for immunotherapy benefit^1,22^. With ICI frequently given together with chemotherapy, ICI biomarker studies require control arms to ensure specificity.

In conclusion, our study describes the peripheral immune landscape of patients undergoing immunotherapy treatment and provides new insights into potential biomarkers associated with therapy outcomes, though further validation is needed. The high-resolution data generated here, coupled with TCR sequences, offers a rich resource and reference for future studies examining peripheral immune biomarkers in breast cancer. Blood-based biomarkers present a unique opportunity for real-time monitoring of patient responses. As blood collection and processing become more standardized for clinical trials and patient care, developing a panel involving circulating tumor DNA/cells and PBMCs will eventually help create an easily accessible tool to predict patient responses to therapy.

## Methods

### Patient-derived peripheral blood samples

Longitudinal peripheral blood samples in this study were from early-stage HR+ breast cancer patients enrolled in the DFCI protocol #16–466 [NCT02999477, manuscript in review; citation to be added]. Participants were randomized 1:1 to receive an upfront 2-week window of nab-paclitaxel (Arm A) or pembrolizumab (Arm B). Patients randomized to Arm A received nab-paclitaxel 125 mg/m^2^ via intravenous (IV) infusion once per week for 2 weeks. Patients randomized to Arm B received one dose of pembrolizumab 200 mg via IV infusion. After the 2-week window, all patients in Arms A and B received weekly nab-paclitaxel 125 mg/m^2^ in combination with pembrolizumab 200 mg once every 3 weeks, for a total planned neoadjuvant therapy course of 12 doses of weekly nab-paclitaxel 125 mg/m^2^ and five doses of pembrolizumab 200 mg administered once every 3 weeks for all patients, regardless of arm. Additional neoadjuvant chemotherapy was allowed at the investigator's discretion in cases of incomplete clinical response to protocol therapy with nab-paclitaxel plus pembrolizumab. To avoid treatment arm covariate, only patients from Arm A were selected for the analysis. Peripheral blood mononuclear cells (PBMCs) were collected at baseline, after 2 weeks of nab-paclitaxel (post-chemo), after one round of nab-paclitaxel + pembrolizumab (post-ICI), and before surgery (endpoint). PT5 and PT11 received additional neoadjuvant adriamycin/cyclophosphamide prior to surgery. All participants were at least 18 years old, with an ECOG performance status of 0 or 1, and provided written informed consent prior to engaging in any study procedures. The study (NCT02999477) was approved by the institutional review board of Dana-Farber/Harvard Cancer Center and was conducted in accordance with the principles of the Declaration of Helsinki.

### Patient tumor molecular subtyping

The BluePrint 80-gene signature (which classifies tumors as Luminal-type, HER2-type, or Basal-type) were performed at Agendia, Inc. (Irvine, CA, USA) on RNA extracted from FFPE tumor tissue.

### Single-cell RNA/TCR sequencing

PBMCs were isolated from EDTA collection tubes, processed by Ficoll gradient, and cryopreserved in 10% dimethylsulfoxide and 90% fetal bovine serum. Thawed PBMCs were depleted of non-viable cells using a dead cell removal kit (Miltenyi, cat no: 130-090-101). Samples were multiplexed in sets of 3 using TotalSeq-C0251/0252/0253 hashtag antibody, clone LNH-94; 2M2 (Biolegend, cat no: 394661, 394663, and 394665). Each sample (targeting 10,000 – 15,000 cells/sample) was processed for single-cell 5’ RNA sequencing utilizing the 10X Chromium system. Libraries were prepared using P/N 1000006, 1000080, and 1000020 following the manufacturer’s protocol. The libraries were sequenced using the NovaSeq 6000 with 150 bp paired-end reads. Illumina’s Real-Time Analysis software (version 2.4.11; Illumina) was used for base calling, and analysis was completed using 10X Genomics Cell Ranger software v2.1.1.

### Data analysis

Data were analyzed in R using *Seurat*^23^. Hashtag oligonucleotides were deconvoluted using HTODemux, with the positive quantile set at 0.85. Next, samples were merged, and all cells were scored for mitochondrial gene expression. Data were transformed using *SCTransform*, regressing against mitochondrial gene expression. Dimensional reduction was performed using *Seurat* integration to minimize batch effects. After initial dimensionality reduction using principal component analysis (PCA), we computed uniform manifold approximation and projection (UMAP) embeddings using the top principal components to capture and depict major sources of variation in the different cell types. General immune cell subtypes were imputed using *scPred*^24^. Six CD8 T cell clusters were generated with *FindClusters* function at a resolution of 0.6 after removing all CD8 T cells without TCR information. Monocyte clusters were generated by setting FindClusters resolution at 0.4. Cell subtype identification was given based on the top ten differentially expressed genes in each cluster. Data aggregation was performed using the *AggregateExpression* function in *Seurat* and further analyzed by DESeq2^25^. Single-sample gene set enrichment analysis (ssGSEA) was performed using the GSVA R package on aggregated expression profiles. This method calculates an enrichment score for each sample and gene set, reflecting the degree to which genes in a predefined set are coordinately up- or down-regulated within that sample. We used the Human MSigDB hallmark gene sets to derive sample-wise activity scores for major cellular processes^26^.

### Tumor TCR sequence extraction

Bulk RNA sequencing result from the patient’s baseline tumor biopsy was previously published [manuscript in review]. The alignment and assembly of the RNA-seq reads and the exporting of the clonotypes were conducted using MiXCR^27^ using default parameters based on the developers’ instructions (https://github.com/milaboratory/mixcr/). In brief, sequencing reads were aligned to reference V, D, J, and C genes of T cell receptors. Then, the aligned reads were assembled to extract CDR3 gene regions. Finally, the clonotypes were exported in a human-readable/parsable tab-delimited text file format.

### Statistical analysis

All statistical analyses were performed in R v.4.1.1. All single-cell statistical analyses were calculated in R using the Seurat package. For differential gene expression analysis, the FindMarker function was applied with a log fold change threshold set to 0.5 and *p*adj threshold set to 10e-5. To calculate the Hallmark gene set score, the EnrichIt function from the Escape package was used. For comparison within the patient or tumor, a paired *t*-test or nonparametric testing was used. For longitudinal comparisons, a paired Wilcox test was used. For multiple comparisons, ANOVA was accompanied by a post hoc Tukey test or a Kruskal–Wallis test with a post hoc Dunn test.

## Supplementary information

Supplementary Information

## Data Availability

Fastq files and processed scRNAseq objects have been submitted to GEO and are under review. GEO accession number: GSE300475.

## Code availability

This manuscript does not report any original code used for the analysis.
