# Supplementary material for: Dynamic single-cell systemic immune responses in immunotherapy-treated early-stage HR+ breast cancer patients
Source: NPJ Breast Cancer. 2025 Jul 3;11:65. doi: 10.1038/s41523-025-00776-1 (PMC12229575; doi:10.1038/s41523-025-00776-1)
Supplement: Supplementary file 1 — Supplementary Information [file 41523_2025_776_MOESM1_ESM.pdf]

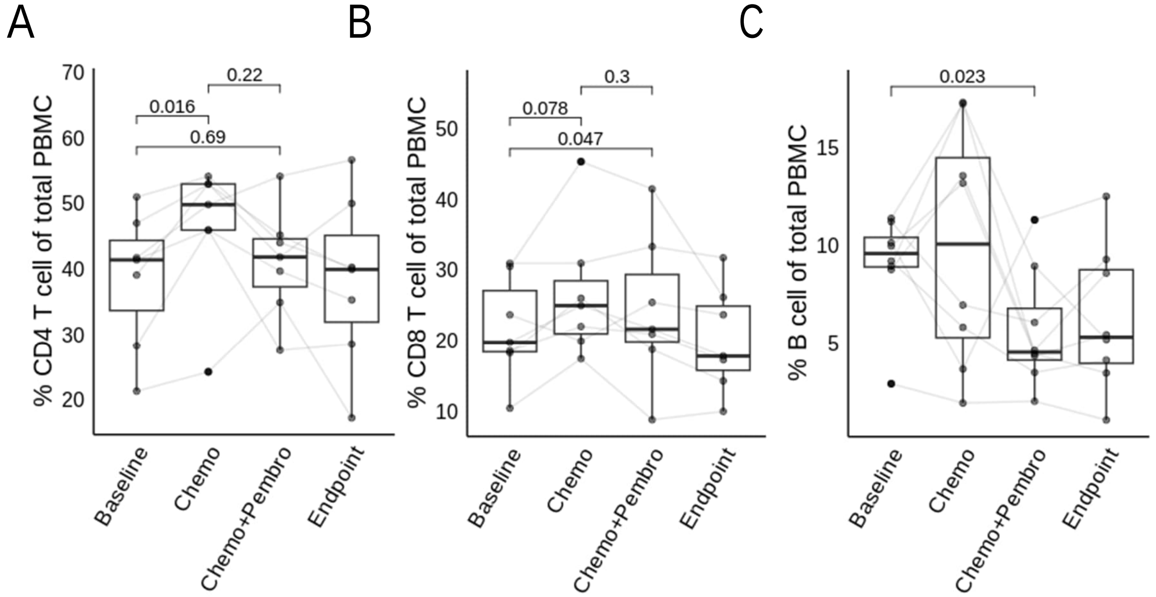

Supplement Figure 1: Temporal dynamic of (A) CD4 T cell, (B) CD8 T cell, (C) B cell was measured across treatment regimen, paired Wilcoxon test performed across timepoints

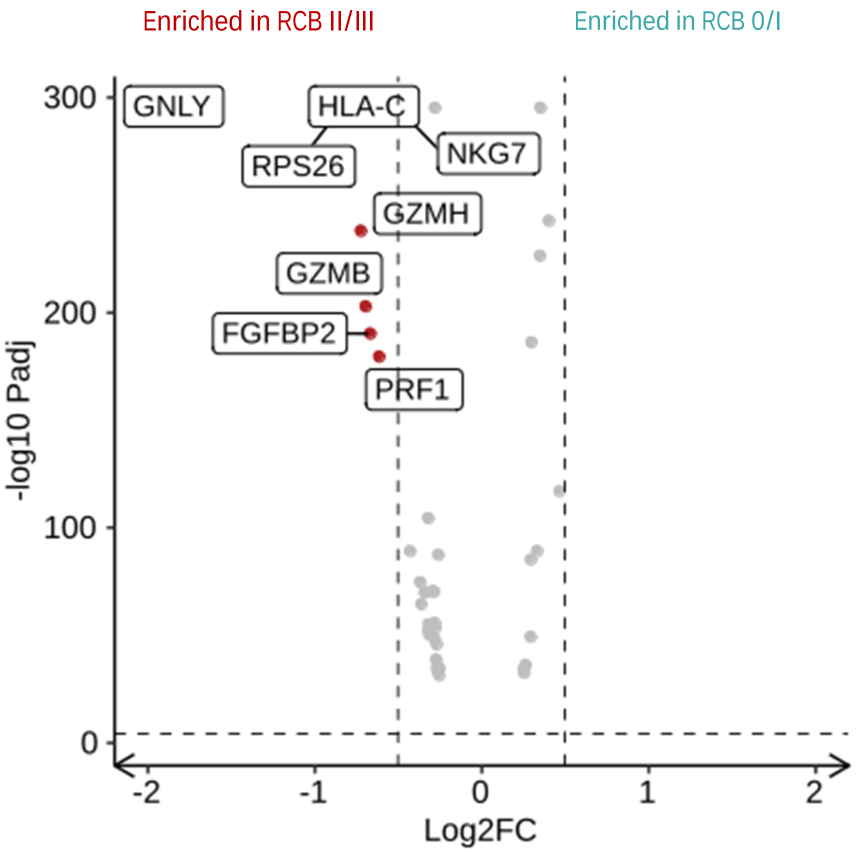

Supplement Figure 2: Differential gene expression analysis performed on CD8 T cell between RCB0/I and RCB II/III patients, log2FC cutoff at 0.5, padj cutoff at 1e-5

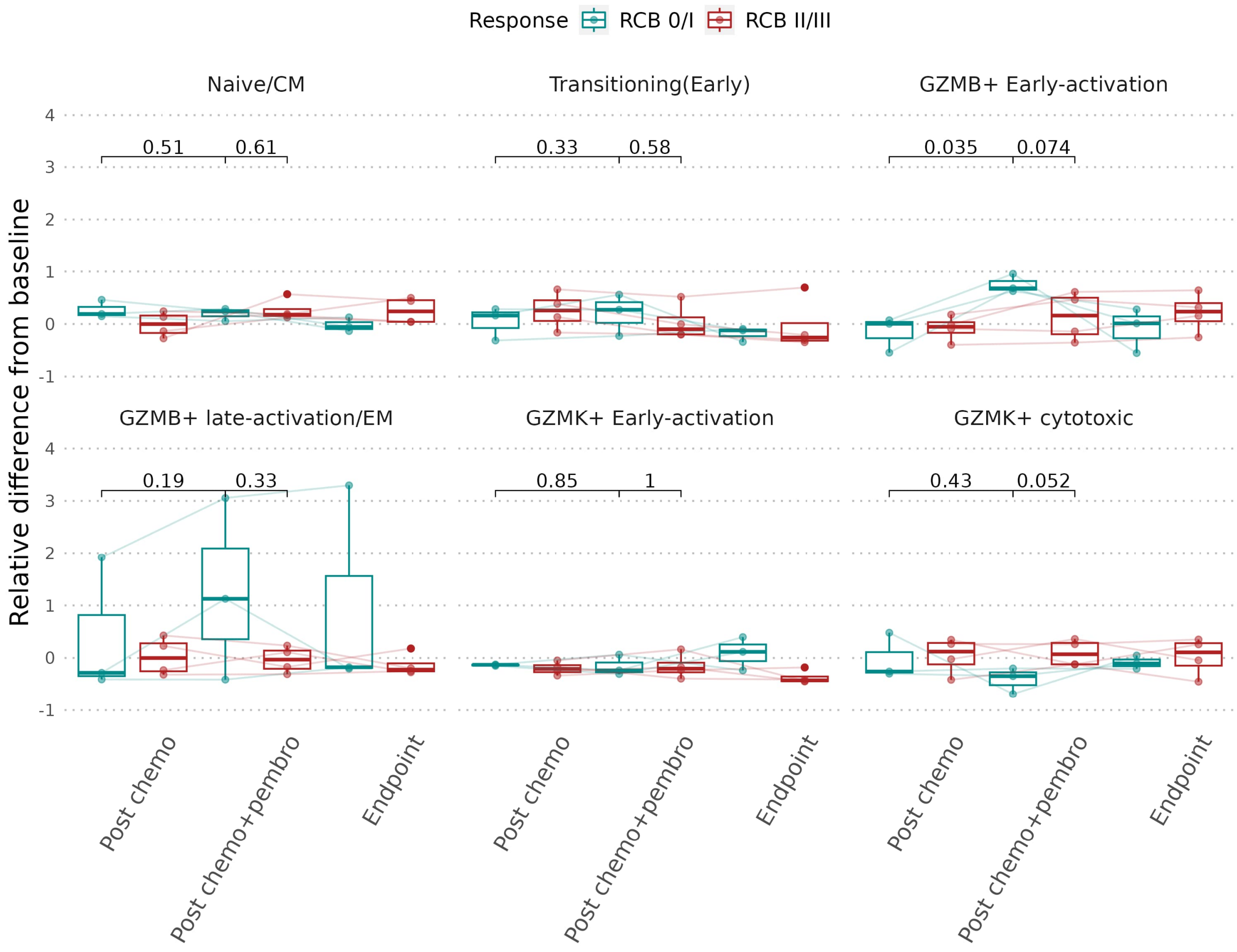

Supplement Figure 3: Post therapy dynamic of each CD8 T cell subtypes was measured as relative changes to baseline. Comparison was performed between post chemo RCB 0/I vs post chemo+pembro RCB0/I patients and post chemo+pembro RCB0/I vs post chemo+pembro RCBII/III patients

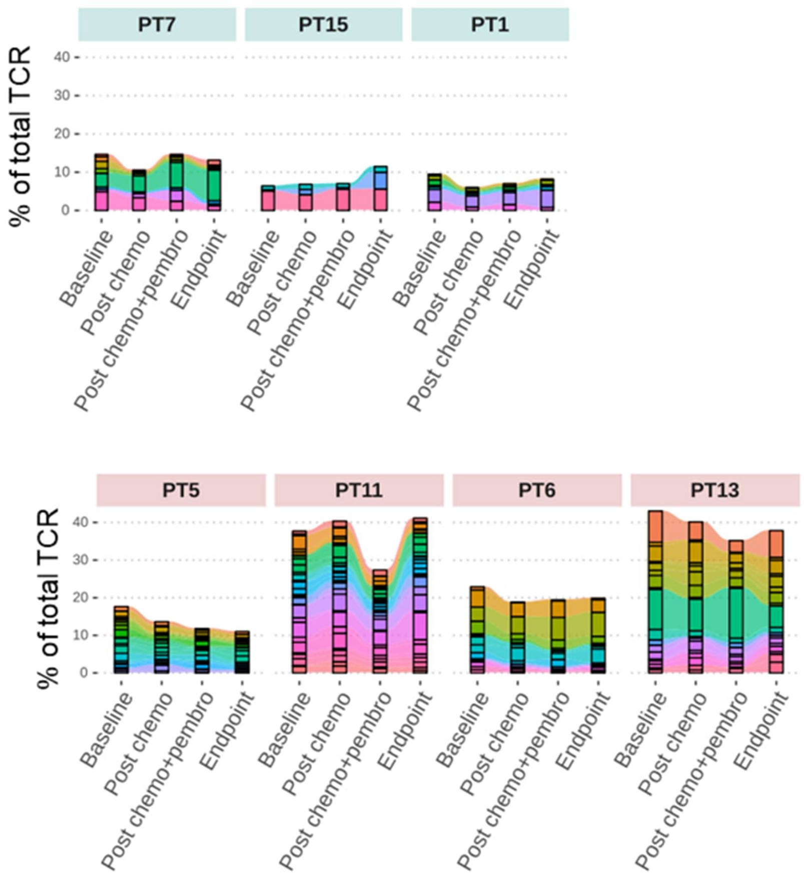

Supplement Figure 4: Temporal dynamic of shared, clonal TCR tracked for individual patients through the course of treatment

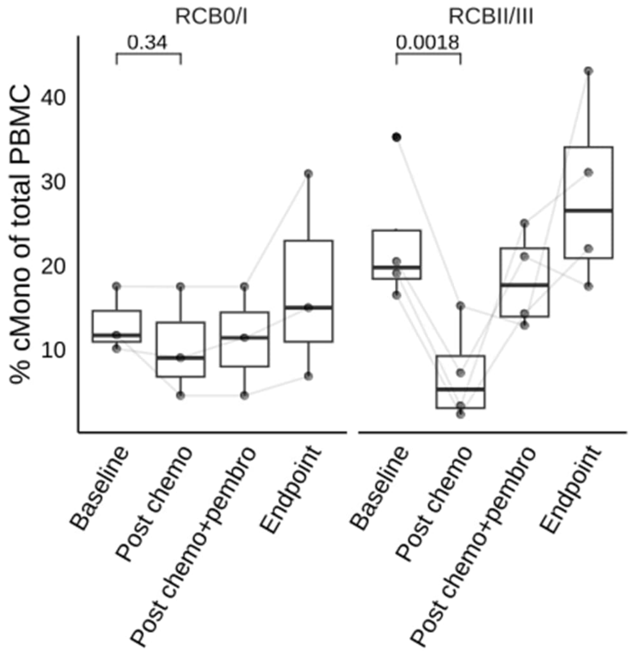

Supplement Figure 5: Abundance of classical monocytes measured longitudinally, paired Wilcoxon test performed.
